# A comparison of hydrophobic polyurethane and polyurethane peripherally inserted central catheter: results from a feasibility randomized controlled trial

**DOI:** 10.1186/s13063-020-04699-z

**Published:** 2020-09-14

**Authors:** Nicole C. Gavin, Tricia M. Kleidon, Emily Larsen, Catherine O’Brien, Amanda Ullman, Sarah Northfield, Gabor Mihala, Naomi Runnegar, Nicole Marsh, Claire M. Rickard

**Affiliations:** 1 Cancer Care Services, Royal Brisbane and Women’s Hospital, Butterfield Street, Herston, Queensland 4029 Australia; 2 Alliance for Vascular Access Teaching and Research, Menzies Health Institute Queensland, Griffith University, Nathan, Queensland 4111 Australia; 3 School of Nursing, Queensland University of Technology, Kelvin Grove, Queensland 4059 Australia; 4 Institute of Health and Biomendical Institute to Healthcare Transformation, Queensland University of Technology, Kelvin Grove, Queensland 4059 Australia; 5 Children’s Hospital Queensland, South Brisbane, Queensland 4101 Australia; 6 Nursing and Midwifery Research Centre, Royal Brisbane and Women’s Hospital, Herston, Queensland 4029 Australia; 7 School of Nursing and Midwifery, Griffith University, Nathan, Queensland 4111 Australia; 8 School of Medicine, Griffith University, Gold Coast, Queensland 4222 Australia; 9 Centre for Applied Health Economics, Menzies Health Institute Queensland, Griffith University, Nathan, Queensland 4111 Australia; 10 Infection Management Services, Princess Alexandra Hospital, Woolloongabba, Queensland 4102 Australia; 11 PA-Southside Clinical Unit, Faculty of Medicine, University of Queensland, Brisbane, Queensland 4102 Australia

**Keywords:** Feasibility, Hydrophobic polyurethane, Peripherally inserted central catheter (PICC), PICC failure, Pilot randomised controlled trial, Polyurethane

## Abstract

**Background:**

To evaluate the feasibility of an efficacy trial comparing a hydrophobic polyurethane peripherally inserted central catheter (PICC) with a standard polyurethane PICC.

**Methods:**

This pilot randomised controlled trial (RCT) was conducted between May 2017 and February 2018. Adult participants (*n* = 111) were assigned to hydrophobic polyurethane PICC with proximal valve (intervention) or a polyurethane PICC with external clamp (standard care). Primary outcome was trial feasibility including PICC failure. Secondary outcomes were central line-associated bloodstream infection, local infection, occlusion, thrombosis, fracture and dislodgement, phlebitis, local or systemic allergic reaction, and PICC dwell time.

**Results:**

All feasibility outcomes were achieved, apart from eligibility criteria. In total, 338 patients were screened, 138 were eligible (41%), and of these 111 were randomised (80%). Patients received the allocated PICC in 106 (95%) insertions. No patients withdrew from the study and there was no missing data. PICC failure was 24% (13/55) in the intervention group and 22% (12/55) in the standard care group (*p* = 0.820). PICC failure per 1000 PICC days was 16.3 in the intervention group and 18.4 in the control group (*p* = 0.755). The average dwell time was 12 days in the intervention and 8 days in the control group.

**Conclusions:**

This study demonstrates the feasibility of an efficacy trial of PICC materials in an adult population, once adjustments were made to include not only in-patients, but also patients being discharged to the Hospital in the Home service.

**Trial registration:**

Australia and New Zealand Clinical Trials Registry ACTRN12616001578493. Prospectively registered on 16 November 2016. The trial protocol was published a priori (Kleidon et al., Vasc Access 3:15–21, 2017).

## Background

Peripherally inserted central catheters (PICCs) are used for intravenous (IV) fluids, medications, and blood products and for blood sampling to prevent frequent phlebotomy [1, 2]. PICCs are the most frequently inserted central venous access device outside of the intensive care unit [3], and their appropriate use [4, 5] enables treatment in diverse settings, including inpatient, outpatient, and community-based [2]. Unfortunately, about one third of PICCs fail prior to completion of treatment [6], often necessitating removal and replacement [7], due to mechanical (blockage, dislodgement, vein thrombosis, rupture) or infective (local or bloodstream infections) complications [6]. PICC complications reduce patient satisfaction, prolong hospitalisation, increase healthcare costs, and risk mortality [8–12].

PICC material and designs have evolved from the first silicone PICCs with external clamps in the 1970s [13] to modern materials and characteristics which include the following: trimmable or un-trimmable catheters, silicone or polyurethane (power injectable and non-power injectable), and anti-microbial or heparin bonding [14]. PICCs are available in various sizes (1 to 6 Fr), configured with single or multiple lumens, open- or closed-ended, with or without external clamps [14]. Despite this abundance of choice, there is a paucity of evidence comparing PICC features. In a recent scoping review [15] of 178 randomised controlled trials (RCTs) in central venous access devices in the past decade, only five studies compared PICC materials [16–20] and two PICC types [21, 22].

A recent addition to the market, BioFlo® is a hydrophobic polyurethane PICC with a surface-modifying macromolecule (Endexo®) that enhances the biocompatibility of medical devices [23]. This durable surface modification occurs during the extrusion moulding manufacturing process. A small amount of polymer/macromolecule is added to the polyurethane/carbothane® to provide hydrophobic properties to the PICC [23, 24]. Earlier unpublished pre-clinical data suggested the Endexo® technology suppressed procoagulant conformation, reduced platelet adhesion, inhibited platelet activation in the presence of blood, and reduced bacteria adhesion and encrustation in the presence of bacteria [23]; however, no anti-infective claim is made by the BioFlo® manufacturers [25]. In addition to surface modification, BioFlo® has a direction-specific pressure-activated safety valve situated at the proximal hub of the PICC, designed to reduce retrograde blood flow into the PICC during normal venous pressures [25]. This technology demonstrates some effectiveness at reducing thrombus accumulation in laboratory settings, but its clinical effectiveness is less established [14].

A recent pilot RCT in paediatric patients [26] compared a polyurethane, power-injected PICC with an external clamp to a hydrophobic polyurethane PICC (BioFlo®). Failure was halved in the hydrophobic polyurethane group (8/72; 11%) compared to standard care (16/74; 22%) (*p* = 0.087), which when expressed as an incident rate was 12.6 and 7.3 per 1000 PICC days (incident rate ratio 0.58; 95% confidence interval (CI) 0.21–1.43; *p* = 0.172). However, this has not been tested in the adult population. Therefore, the primary aim of our feasibility RCT was to compare the effectiveness of a hydrophobic polyurethane PICC with a standard polyurethane PICC to prevent PICC failure. We hypothesised that conducting an RCT would be feasible.

## Methods

### Design

This pilot parallel RCT with 1:1 allocation compared a hydrophobic polyurethane PICC with pressure-activated proximal valve to a polyurethane PICC with external clamp.

### Study setting

This trial recruited between May 2017 and February 2018 at the Royal Brisbane and Women’s Hospital (RBWH) in Brisbane, Australia, a large quaternary hospital. During the study, PICCs at this hospital were inserted in the Department of Medical Imaging, which provided a specialist diagnostic imaging and radiology service, supporting the care and treatment of patients. Patients with a PICC were either be treated in a traditional hospital ward or in the Hospital in the Home (HITH) service, which allowed patients to receive their hospital intravenous treatment in their own homes.

### Sample

The target sample size was 110 participants, 50 participants per group plus 10% for potential attrition, as determined by pilot trial sample size recommendations [27]. Patients were consecutively recruited and randomised if they met the inclusion criteria: PICC to be inserted in the Department of Medical Imaging for fluid or medication administration, predicted hospital inpatient admission > 24 h, 18 years or older, and provided informed consent. Due to slow recruitment, the inclusion criteria were expanded to include patients who were transferred to the HITH service within 24 h of PICC insertion. Patients were excluded if they had a current bloodstream infection (classified as a positive blood culture within 48 h prior to PICC insertion); had an allergy to the study product; the PICC was to be inserted through diseased, burned, or scarred skin; the PICC was inserted in other departments or units in the hospital; they could not provide consent without an interpreter; had a previous enrolment in the study; or had an existing central venous access device including pulmonary artery catheters.

Participants were randomised to receive either (1) standard care—polyurethane, power-injectable PICC with external clamp (Arrow International, Reading, Pennsylvania)—or (2) the intervention—hydrophobic polyurethane PICC with a proximal valve (BioFlo® with Endexo® and PASV®, AngioDynamics Inc., Queensbury).

### Outcomes

#### Primary outcomes

##### Feasibility

The primary outcome was feasibility of conducting a large, definitive RCT comparing hydrophobic polyurethane PICC with simple polyurethane to prevent PICC failure and complications in adults. Feasibility was assessed as a composite analysis of elements [27–29] of the following: (i) eligibility (> 70% of screened patients were eligible), (ii) recruitment (> 70% of eligible patients agreed to enrol), (iii) retention and attrition (< 15% of participants are lost to follow-up or withdrew from the study), (iv) protocol adherence (> 80% of participants received their randomly assigned study product), (v) missing data (< 10% of endpoint data were missed during data collection), and (vi) patient and healthcare professional satisfaction and acceptability. Patient satisfaction with the PICC was collected as pain at removal on an 11-point Likert scale (0 being no pain and 10 being worst imaginable pain). PICC inserters were asked to rate their levels of satisfaction with insertion equipment (0 being very dissatisfied and 10 being very satisfied) and ease of PICC insertion (0 being very difficult and 10 being very easy) on an 11-point Likert scale. For staff satisfaction and acceptability, nurses were asked to rate their difficulty during PICC removal on an 11-point Likert scale (0 being no difficulty and 10 being significant difficulty); and (vii) sample size estimates for a definitive trial.

##### PICC failure

PICC failure was defined as the following complications associated with PICC removal: (i) central line-associated bloodstream infection (CLABSI) [30], (ii) local infection [30], (iii) device occlusion [31, 32], (iv) venous thrombosis [33], and (v) PICC fracture or dislodgement [31, 34]. The outcome of device failure was an objective measure, assigned by clinical staff (not research staff or investigators). The published protocol by Kleidon and colleagues [35] defines PICC failure in further detail.

#### Secondary outcomes

In addition to individual complications (CLABSI, local infection, occlusion, thrombosis, fracture, and dislodgement), secondary outcomes included the following: (i) phlebitis, defined as any sign of chemical, mechanical, or infective phlebitis, determined by patient complaint of pain and nurse examining PICC site; (ii) safety endpoints (local or systemic allergic reactions); and (iii) PICC dwell time in days.

##### Study procedures

A research nurse (ReN) screened for eligible patients. Patients who agreed to participate were randomised via computer-generated randomisation immediately before PICC insertion via a web-based service (https://randomisation.griffith.edu.au/) to ensure allocation concealment until study entry. Patients were randomly assigned in a 1:1 ratio with randomly varied block sizes of two and four. The ReN provided the randomised study product to the inserting clinician. Data were collected using Research Electronic Data Capture [36]. Demographic and clinical data were collected at recruitment. The ReN visited inpatients twice weekly and the HITH nurse visited daily until the PICC was removed due to treatment completion, PICC failure, or until 4 weeks (maximum follow-up). Primary and secondary outcomes were collected by the ReN from the medical records, clinical staff, and patient assessment. Outcome data were collected at 48 h post PICC removal. The infection control physician (adjudicating infection outcomes), radiologist (adjudicating thrombosis outcomes), and data analyst were blinded to the study allocation. Infection and mortality data were collected at 48 h post PICC removal. Participants were followed for 4 weeks, unless the PICC was removed earlier. It was not possible to blind the PICC inserters, patients, or healthcare professionals caring for the patients enrolled in the study.

##### PICC procedures

Prior to the study, the intervention PICC was not used in the hospital. Pre-study training for PICC inserters, inpatient, and HITH staff was provided by the manufacturer in the month preceding study commencement and for the first month after recruitment of the first patient. The ReN provided ongoing training and support for PICC inserters and clinical staff caring for the PICC.

PICCs were inserted by a registered nurse, radiographer, or medical officer; experience ranged from novice to expert. PICC insertion occurred in either digital fluoroscopy or the angiography suite. Asepsis was maintained using maximal barrier precautions including use of 2% chlorhexidine gluconate in 70% isopropyl alcohol skin preparation (Soluprep; 3M) [37]. Ultrasound was used for all initial venous assessment and initial vein puncture. A modified Seldinger technique was used for the remainder of the PICC insertion. Fluoroscopy was used to confirm optimal catheter tip placement in the lower third of the superior vena cava or cavo-atrial junction. When necessary, 10 mL of iodinated contrast and digital subtraction angiography was used to assist accurate tip placement during complicated PICC insertion. Dressing and securement were achieved with a chlorhexidine impregnated foam disc (Biopatch®; Ethicon, New Jersey), a sterile semi-permeable, adhesive transparent dressing (IV3000 10 × 12cm; Smith and Nephew; Hull), and a stabilisation device (StatLock® PICC Plus Stabilization Device; Bard, Georgia) [37].

Post insertion care and maintenance was standardised with the PICC dressing and needleless connectors (SmartSite™; BD, Utah) replaced weekly or as required if soiled or lifting [38]. Inpatients who had continuous infusions had their IV administration sets replaced every 72 h, excluding blood products or chemotherapy, which were replaced on completion of the infusion. Intermittent infusions were flushed and locked with 0.9% sodium chloride. Complications were identified and monitored by the treating team. Occlusion was managed with a thrombolytic agent (e.g. urokinase). The decision to take blood cultures and remove PICCs was made by the medical officer treating the patient. HITH patients were visited daily to have their elastomeric device (Infusor™; Baxter, Illinois) and antibiotic replaced. If HITH patients required treatment to manage a PICC complication, such as administration of a thrombolytic agent, they were re-admitted to the ward.

### Statistical analyses

Data were exported to Stata 15 for cleaning and analysis. Data cleaning of outlying figures and missing and implausible data were undertaken prior to analysis. Missing data were not imputed. The patient was the unit of measurement, and all randomly assigned patients were analysed on an intention-to-treat basis [39]. Descriptive statistics (frequencies and percentages) were used to ascertain the primary outcome of feasibility for the larger trial. Incidence rates (per 1000 PICC days) and rate ratios, including 95% confidence intervals, were calculated. The comparability of groups at baseline was described across demographic, clinical, and device characteristics. Kaplan-Meier survival curves (with log-rank tests) were used to compare PICC failure between study groups over time. Associations between failure and baseline characteristics were described by calculating hazard ratios. Multivariable Cox regression was not performed due to the relatively low number of outcomes. *p* values of < 0.05 were considered statistically significant.

## Results

### Participant and PICC characteristics

The participant and PICC characteristics are described in Table 1. Over 90% of patients recruited to this study were medical or surgical patients. Demographic and PICC insertion characteristics were balanced between the groups. The PICC inserters were more experienced at inserting the standard care PICC: in only 1/52 (2%) PICC insertions had the PICC inserter never inserted a standard care PICC compared to 16/56 (29%) in the intervention group, and three quarters of the PICC inserters (39/52; 75%) had inserted more than 11 standard care PICCs compared to 3/56 (5%) in the intervention group.

**Table 1:** Participant and insertion characteristics at insertion

	***n***	Intervention	Control	Total
Group size^a^	111	56 (50)	55 (50)	111 (100)
Female gender	111	28 (50)	29 (53)	57 (51)
Age (years)^b^	111	59 (15)	62 (16)	61 (16)
Body mass index^b^	111	28 (10)	31 (16)	30 (13)
Comorbidities	111			
None		2 (4)	1 (2)	3 (3)
One		7 (12)	8 (15)	15 (14)
Two		9 (16)	5 (9)	14 (13)
Three		7 (12)	6 (11)	13 (12)
Four or more		31 (55)	35 (64)	66 (59)
History of^c^				
Clot	109	17 (30)	17 (32)	34 (31)
Antithrombotic therapy	108	46 (84)	48 (91)	94 (87)
Diagnosis at admission	111			
Surgical		39 (70)	35 (64)	74 (67)
Medical		12 (21)	15 (27)	27 (24)
Other		5 (9)	5 (9)	10 (9)
Infection at time of recruitment	111	38 (68)	31 (56)	69 (62)
Wound at time of recruitment	111	35 (62)	27 (49)	62 (56)
Side of insertion: non-dominant arm	110	33 (60)	35 (64)	68 (62)
PICC placement:	110			
Basilic		46 (84)	40 (73)	86 (78)
Brachial		8 (15)	12 (22)	20 (18)
Other		1 (2)	3 (5)	4 (4)
Catheter size:	110			
5 Fr		54 (98)	53 (96)	107 (97)
4 Fr		1 (2)	2 (4)	3 (3)
Lumens	110			
Two		54 (98)	53 (96)	107 (97)
One		1 (2)	2 (4)	3 (3)
Difficult insertion	111	20 (36)	13 (24)	33 (30)
Number of attempts	110			
One (first time success)		40 (71)	42 (78)	82 (75)
Two		9 (16)	6 (11)	15 (14)
Three or more		7 (12)	6 (11)	13 (12)
Inserted by	110			
Radiographer		27 (49)	26 (47)	53 (48)
Nurse		21 (38)	21 (38)	42 (38)
Doctor		7 (13)	8 (15)	15 (14)
Ease of insertion^d,e^	108	7.5 (5.0–9.0)	10.0 (7.0–10.0)	8.0 (5.0–10.0)
Satisfaction with insertion kit^d,e^	108	7.0 (3.0–8.0)	10.0 (10.0–10.0)	9.0 (7.0–10.0)
PICC inserter level of experience	108			
No history of insertion		16 (29)	1 (2)	17 (16)
One to ten devices inserted		37 (66)	12 (23)	49 (45)
11 or more devices inserted		3 (5)	39 (75)	42 (39)

Frequencies and column percentages shown unless noted otherwise

*n* number of non-missing observations

^a^Row percentages shown

^b^Mean (standard deviation)

^c^Multiple responses possible per participant

^d^Median (25–75th percentiles) shown

^e^On a 0 to 10 scale, 0 = worst and 10 = best

### Feasibility outcomes

**Fig. 1:**
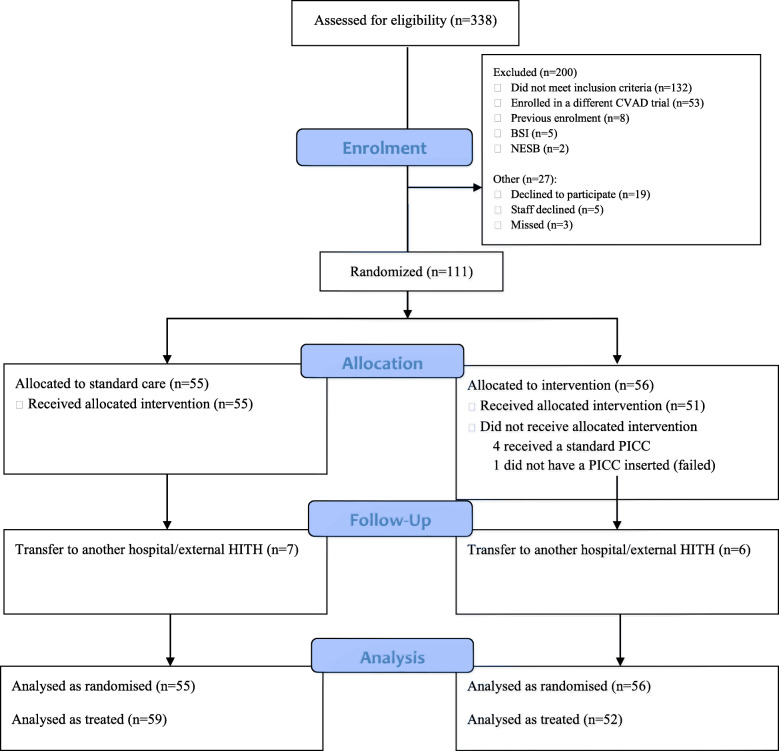
CONSORT 2010 flow diagram

Apart from eligibility criteria, all feasibility outcomes were achieved. In total, 338 patients screened, 138 were eligible (41%), and of these 111 were randomised (80%). Figure 1 outlines the reasons for unsuccessful achievement of the eligibility criteria. Additionally, Fig. 1 displays the flow of patients through the study confirming > 80% participants received their randomly assigned study product. Of the five who did not receive the intervention (106/111 received the allocated treatment, 95%), four received the standard care PICC. No patients withdrew from the study; therefore, the retention and attrition criteria of < 15% were met. Thirteen (12%) patients were censored on transfer to another hospital (seven in the standard care and six in the intervention group). There was no missing data for the primary outcome. The research team did not have ethical approval to request clinical information for these patients transferred to another hospital.

PICC inserter satisfaction with ease of insertion with the insertion equipment was rated higher in the standard care group (10.0 in the standard care group and 7.0 in the intervention) (Table 1). Although the number of insertion attempts was balanced between the groups, there were more difficult insertions in the intervention group (20; 36% in the intervention group compared to 13; 24% in the standard care group). Patient satisfaction and acceptability was collected as pain on PICC removal. Of the twenty-six patients asked, no one reported pain at removal (Table 2).

**Table 2:** End points

	***n***	Intervention	Control
Group size^a^	111	56 (50)	55 (50)
Reason for study completion	110		
Removed		41 (75)	42 (76)
Patient transferred		6 (11)	7 (13)
4 weeks completed		7 (13)	5 (9)
Patient deceased		1 (2)	1 (2)
Reason for removal^b^	83		
tx completed, no device complications		27 (66)	29 (69)
tx incomplete, device complications		12 (29)	11 (26)
tx completed, device complications		1 (2)	1 (2)
Transferred, no device complications		1 (2)	1 (2)
Complications (resulting in failure)^c^			
Any complication	110	13 (24)	12 (22)
PICC-associated BSI, suspected	25	6 (46)	5 (42)
Dislodgement, full	25	4 (31)	7 (58)
Occlusion	25	3 (23)	1 (8)
Skin reaction	25	1 (8)	0 (0)
Fracture	25	0 (0)	0 (0)
Suspected thrombus	25	0 (0)	0 (0)
Complications (during tx)^c^			
Any complication	110	25 (45)	20 (36)
Occlusion	45	15 (60)	11 (55)
PICC-associated BSI, suspected	45	8 (32)	3 (15)
Dislodgement, partial	45	7 (28)	4 (20)
PICC-associated thrombosis, suspected	45	1 (4)	1 (5)
Other	45	4 (16)	3 (15)
Serious adverse events^c^			
Any type	110	5 (9)	6 (11)
Positive blood culture	11	3 (60)	3 (50)
Unplanned admission to ICU	11	2 (40)	2 (33)
Death	11	1 (20)	2 (33)
Infection (baseline or during tx)^c^			
Any type	110	45 (82)	37 (67)
Wound	82	14 (31)	12 (32)
Urinary	82	8 (18)	9 (24)
Bone	82	4 (9)	7 (19)
Faecal/gastrointestinal	82	7 (16)	3 (8)
Respiratory	82	5 (11)	4 (11)
Skin/cellulitis	82	7 (16)	1 (3)
Other	82	11 (24)	13 (35)
Unknown	82	4 (9)	5 (14)
Confirmed BSI classifications (count)^c^			
LCBI (common commensal)	110	3 (5)	0 (0)
CLABSI	110	0 (0)	2 (4)
MBI-LCBI	110	1 (2)	0 (0)
Thrombus, confirmed	110	1 (2)	0 (0)
Pain at removal (0 = worst, 10 = none)^d^	26	0.0 (0–0)	0.0 (0–0)
Outpatient/HITH tx	110	19 (35)	14 (25)

Frequencies and column percentages shown unless noted otherwise

*ICU* intensive care unit, *PICC* peripherally inserted central catheter, *BSI* bloodstream infection, *incl.* including, *HITH* hospital in the home, *CLABSI* central line associated bloodstream infection, *MBI-LCBI* mucosal barrier injury laboratory confirmed bloodstream infection, *LCBI* laboratory confirmed bloodstream infection, *tx* treatment

^a^Row percentages shown

^b^Denominator was the number of observations with device removed

^c^Multiple outcomes per device possible

^d^Median (25th/75th percentiles shown)

### PICC failure

**Fig. 2:**
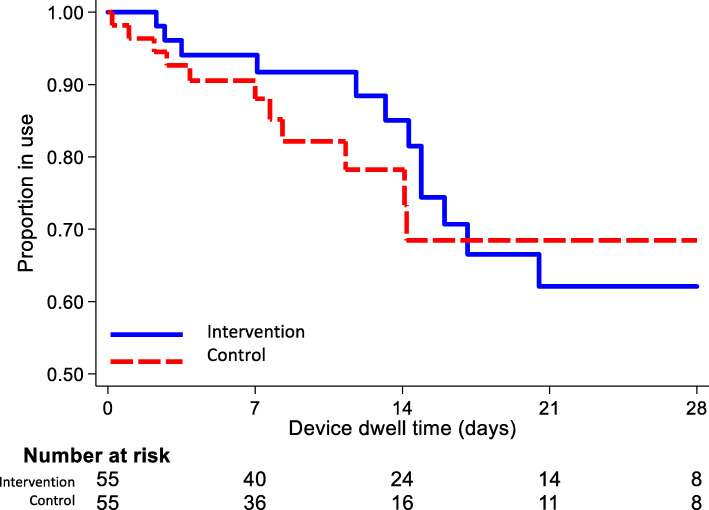
Kaplan-Meier survival curve

In total, 25 out of 110 patients (23%) experienced PICC failure: of these, two patients (one in each group) had completed their treatment and 23 had not. Presented as absolute numbers, 13/55 (24%) PICCs failed in the intervention group and 12/55 (22%) in the standard care group (*p* = 0.820). The risk ratio was 1.08 (0.54–2.16), and the risk difference was 1.8% (− 13.8–17.5). PICC failure per 1000 PICC days was 16.3 (95% CI 9.5–28.1) in the intervention group and 18.4 (95% CI 10.5–32.5) in the control group (*p* = 0.755). Failure was most commonly due to dislodgement, followed by occlusion. See Tables 2 and 3, also Fig. 2. The average dwell time was 12 days in the intervention and 8 days in the control group (Table 3). During the study, six adverse events were recorded: one skin reaction (in the intervention group), four patients were transferred to the intensive care unit (two in each group), and three patients died (one in the intervention and two in the standard care group). In all cases, the patients’ deterioration was not related to the PICC.

**Table 3:** Failure rates and survival analysis

	***n***	Intervention (***n*** = 55)	Control (***n*** = 55)	***p*** value
PICC failure	110	13 (24%)	12 (22%)	0.820^a^
Dwell time (days)^b^	110	12 (5–21)	8 (5–15)	0.175^c^
Device days	110	797	651	–
Incidence rate (per 1000 PICC days)^d^		16.3 (9.5–28.1)	18.4 (10.5–32.5)	0.755^e^
Incidence rate ratio		0.89 (0.37–2.12)	Reference	

hyphen = not calculated

^a^Chi-squared test

^b^Median (25–75th percentiles) shown

^c^Wilcoxon rank-sum test

^d^Rate and 95% confidence interval shown

^e^Cox univariable regression

## Discussion

This study demonstrates the feasibility of an efficacy trial of PICC materials in an adult population, once adjustments were made to include patients to be discharged to the HITH service. No lead-in time with the intervention PICC was possible outside of the trial; therefore, PICC inserters were inexperienced with the new device. This study included a broad selection of medical/surgical patients who required a PICC for treatment in the hospital and/or as a HITH patient in their own homes. Patients ranged from being ambulant requiring long-term antibiotics to patients with major surgery, such as pelvic exenterations. To further improve eligibility, future trials might consider surrogate decision maker and telephone consent as 21 out of the 338 (6%) patients approached were too confused to consent. Despite these challenges, it is important that an adequately powered trial comparing the effectiveness of hydrophobic PICCs to other PICC technologies is conducted, as PICC failure remains unacceptably high. Previous systematic reviews have highlighted that one-third of PICCs fail [6] with comparable incidence seen in our study. Clinicians and policy makers need to take urgent steps to investigate potential improvements in PICC outcomes since failure disrupts patient treatment due to delay in insertion of a new PICC and multiple PICCs can increase patient complications and decrease the quality of a patient’s experience [8–12]. PICCs are associated with morbid complications, such as CLABSI and deep vein thrombosis; thus, it is essential researchers generate further evidence to guide clinicians to select the most appropriate PICC materials to reduce these potentially fatal adverse events and improve the overall quality of care provided to patients [14, 40].

This pilot RCT compared a hydrophobic polyurethane PICC with proximal valve with a power-injectable polyurethane PICC with external clamp to reduce PICC failure and complications in an adult population. This study followed the same methodology as the paediatric protocol [35] and published study [26] and was reported in line with the CONSORT guidelines. A quarter of PICCs failed (25/110; 23%) before treatment completion. The primary objective of this study was feasibility (rather than reducing PICC failure); and although the incident rate ratio of 0.89 favoured the intervention, PICC failure was not significantly different between the groups (12/55; 22% in the standard care and 13/55; 24% in the intervention group).

To date, six studies, published in peer reviewed journals and as conference abstracts, have compared the BioFlo® PICC with non-hydrophobic PICCs: one paediatric pilot RCT [26], one adult RCT [41], one quasi-experimental clinical evaluation [42], and three retrospective cohort studies [43–45]. With the exception of the paediatric pilot RCT [26], it is difficult to assess the risk of bias in the other studies published to date as they are conference abstracts. All but one [41] study demonstrated an improvement in PICC-associated thrombosis and occlusion with BioFlo®. Musial and Hamad [43] reported in their economic evaluation that despite the reduction in occlusion with BioFlo® PICC, when the increased cost of BioFlo® was compared to cost savings from reduced use of the thrombolytic treatment alteplase (Cathflo Activase, Genentec Inc., San Francisco, CA), there was no economic benefit, but they did not consider costs of lost treatment time and extended inpatient stay. Currently, there is insufficient clinical data to definitively demonstrate the potentially beneficial hydrophobic properties of BioFlo® PICC in clinical practice, thus further large trials have commenced.

Limitations of this study include the length of follow-up of patients. We followed our patients for up to of 4 weeks rather than until PICC removal. This might explain why only four incidences of occlusion resulting in PICC failure occurred (see Table 2). Another limitation was that only patients showing clinical symptoms of thrombus were referred for an ultrasound for confirmation. This may explain the low rate of PICC-associated thrombus in this cohort. Future studies should consider routine ultrasounds as approximately two-thirds of PICC-associated thrombi are asymptomatic [46]. Additionally, it was not possible to blind the PICC inserters, patient or other healthcare professionals. Despite these limitations, intervention fidelity was strong and the potential for reproducibility in future trials through the publication of the trial protocol [35] and reporting of the study and PICC procedures, which allows for generalisability of the study results.

The results of this study should be interpreted with caution as it reports the results of a pilot RCT that recruited 110 patients. A full-scale adequately powered efficacy trial is required to test the statistical hypotheses of the efficacy of a hydrophobic polyurethane PICC with proximal valve compared to a plain polyurethane PICC with external clamp to reduce PICC failure and complications. A multi-centre trial recruiting adults and children would ensure these results are generalizable beyond this single centre and demonstrate external validity and safeguarding internal validity with comparable groups at baseline.

## Conclusions

In conclusion, this study demonstrated that it is feasible to conduct an RCT comparing the efficacy of hydrophobic polyurethane PICCs (with pressure-activated proximal valves) with polyurethane PICCs (with external clamps) in an adult population receiving treatment at a large quaternary referral hospital and/or HITH service. It is important that future studies evaluate cost-effectiveness, including inserter and patient acceptability, to robustly evaluate the impact of different PICC materials on failure. Additionally, this study demonstrated the importance of providing a sufficient lead-in time to train PICC inserters before the commencement of data collection as lack of familiarity with an interventional product could impact study results.

## Data Availability

The datasets generated and/or analysed during the current study are not publicly available due to our Human Research and Ethics Committee approval but are available from the corresponding author on reasonable request.

## Abbreviations

CLABSI: Central line-associated bloodstream infection
HITH: Hospital in the Home
PASV®: Pressure-activated safety valve
PICC: Peripherally inserted central catheter
RBWH: Royal Brisbane and Women’s Hospital
RCT: Randomised controlled trial
ReN: Research nurse
